# Understanding and improving the language of business: How accounting and corporate reporting research can better serve business and society

**DOI:** 10.1007/s11573-023-01158-4

**Published:** 2023-05-20

**Authors:** Rolf Uwe Fülbier, Thorsten Sellhorn

**Affiliations:** 1 Universität Bayreuth, Bayreuth, Germany; 2 Ludwig-Maximilians-Universität München, Munich, Germany

**Keywords:** Accounting research, Digital transformation, Research assessment, Sustainability reporting, Societal relevance, Transparency

## Abstract

Financial accounting, the core of corporate reporting, is often characterized as the ‘language of business’. Over the last roughly 100 years, and using an evolving set of theories, methods, and data, scholarly work in this area has been contributing to our understanding of this language and how to improve it. This paper seeks, first, to characterize the field with a focus on its evolution in the German-speaking area, where, like elsewhere, normative research traditions interested in improving practice have been making way for positivist approaches that seek a detached understanding of ‘what is.’ Second, we discuss the changing users and institutional parameters that are reshaping corporate reporting, followed by our personal view of ‘wicked’ societal problems and challenges that corporate reporting might be able to help alleviate. Finally, we discuss directions in which research might evolve in order to address these issues, in order to make corporate reporting more useful for serving not only economic actors, but also society and the environment more broadly.

## Introduction

Financial accounting as the centerpiece of corporate reporting has been dynamically evolving, shaped by changing business and social environments, in order to fulfill changing objectives. Accounting research has been closely accompanying this development. It has helped to better understand and to improve financial accounting as the language of business. We discuss new challenges to financial accounting, which motivate research contributions in several respects. In particular, we address the question of how today’s challenges are forcing research to move forward if it wants to remain relevant for the further development of financial accounting and corporate reporting.

The metaphor of financial accounting as ‘the language of business’ is ubiquitous.^1^ It implies that financial accounting is a communication device by which senders of information seek to make themselves understood to receivers, by being cooperative (Grice 1975), i.e., “accurate (maxim of truth) and complete (maxim of quantity), while communicating in ways that are relevant to the listener (maxim of relation) and as brief and clear as possible (maxim of manner)” (Bloomfield 2008, p. 434). Practiced in this way, corporate reporting enhances transparency (i.e., quality of information), which comprises disclosure, accuracy, and clarity.^2^

To the extent that the other elements of corporate reporting also follow this intent, the language metaphor extends to corporate reporting as a whole. In this paper, we view financial accounting as a subset of a wider corporate reporting, which includes monetized accounting numbers as well as (financial and sustainability-related) disclosures, and which firms use to inform their external capital providers and other stakeholders about their financial position, performance and enterprise value—as well as its environmental and social impacts. Drawing on Barker and Mayer (2021) and the ‘Group of Five’ prototype (CDP et al. 2020), Table 1 describes the elements of corporate reporting. In terms of information systems (reflected in the columns), corporate reporting consists of financial accounting (i.e., primarily quantified information about the firm’s past transactions and events, expressed in monetary units) and disclosure (i.e., additional, complementary qualitative and quantitative as well as backward- and forward-looking information). Whereas accounting information is typically provided in the firm’s primary financial statements and notes, disclosure is often located in a supplementary management report, or management commentary. Taken together, a firm’s accounting and disclosure form its reporting.

**Table 1:** Elements of corporate reporting (drawing on Barker and Mayer 2021 and CDP et al. 2020)

Information system perspective	Accounting (mostly quantitative, past transactions and events)	Disclosure (qualitative and quantitative, backward- and forward-looking)	Reporting
Financial Materiality (‘Exposure Materiality’)	**I—Financial Accounting** Financial statements and notes prepared under Generally Accepted Accounting Principles (e.g., IFRS Accounting Standards)	**II—Value-relevant Disclosure** Financial and non-financial disclosures, including on the firm’s exposure to sustainability matters that create or erode enterprise value (e.g., prepared under IFRS Sustainability Disclosure Standards, or under ESRS that adopt financial materiality)	**I + II = ** **Financial Reporting**
Environmental-social Materiality (‘Impact Materiality’)	**III—Impact Accounting** Monetized amounts of firms’ environmental and social impacts not captured in Financial Accounting (‘externalities’)	**IV—Impact Disclosure** Financial and non-financial disclosures on the firm’s impacts on social and environmental value (e.g., prepared under ESRS that adopt impact materiality)	**III + IV = ** **Impact** **Reporting**
Double Materiality (either or both)	**I + III = ** **Sustainability Accounting**	**II + IV = ** **Sustainability Disclosure**	**I + II + III + IV = ** **Corporate Reporting**

As the lines in Table 1 indicate, another dimension of corporate reporting is its perspective. Financial accounting and value-relevant disclosure adopt a financial (exposure) materiality (or outside-in) perspective, concentrating upon those transactions, events and expected future risks and opportunities that have the potential to materially affect enterprise value, and which are therefore relevant to financially oriented investors and other providers of capital. On the other hand, impact reporting takes an environmental-social (impact) materiality (or inside-out) perspective, focusing on those activities of the firm that have the potential to materially affect the environment or society, and which are therefore of interest to non-financially oriented capital providers as well as a broad range of other stakeholders. Taken together, we refer to the four quadrants of Table 1 combined as corporate reporting, where quadrants I and III form the (mostly quantitative) accounting, which is increasingly complemented by disclosure (quadrants II and IV).

**Fig. 1:**
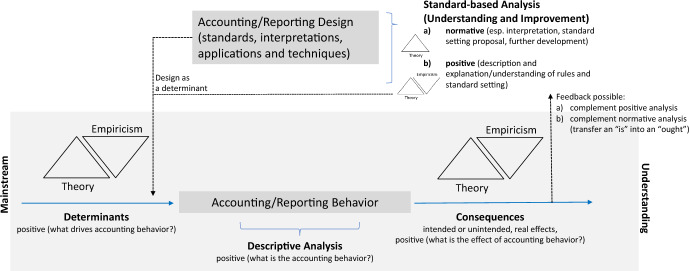
Focus of financial accounting/corporate reporting research

We view corporate reporting research as having evolved to understand and improve the role of corporate reporting as a language that protects stakeholders (primarily providers of capital) by mitigating information asymmetries between corporate managers and these outside stakeholders, as well as among different groups of outside stakeholders (e.g., more versus less sophisticated investors). In terms of *understanding* (‘positive research’), landmark studies too numerous to mention here have sought to establish, theoretically and empirically, the determinants and consequences of corporate reporting behavior. However, earlier research focused—and in some research communities still does—on the conceptual and technical design of standards and on questions of their application. This type of analysis is often normative (prescriptive) in terms of its methodological and epistemological character, especially in that it seeks to *improve* future corporate reporting, rather than understand existing corporate reporting. Figure 1 illuminates these interwoven areas of research. Thus, the institutional level of corporate reporting standards is one important determinant of behavior, just as a better understanding of behavior in turn helps to improve the standardization of corporate reporting.

We start in Sect. 2 by outlining these research traditions from a German-speaking perspective. Considering this historical development at the outset of the paper is important for recognizing the dynamic character of accounting and corporate reporting research, which does not (and must not) remain static, but requires further development in the face of changing conditions. Section 3 describes recent (and not-so-recent) changes in corporate reporting that arguably provide an impetus for research to shift its focus. In Sect. 4, we suggest directions and areas of focus for future corporate reporting research in order to render its insights more useful for addressing important societal problems. In doing so, we also draw on an informal survey of research priorities that we conducted with several senior practitioners and colleagues. Section 5 concludes.

## Where we come from

### Improving and understanding: changing research patterns over time

Financial accounting research as the nucleus of corporate reporting research emerged more than 100 years ago. In Germany and elsewhere in the world, accounting academics primarily developed normative guidelines for conceptualizing, designing and improving financial statements (e.g., Küpper and Mattessich 2005; Mattessich 2008; Fülbier and Gassen 2011; Biondi and Zambon 2013). Although double-entry-bookkeeping techniques have been used in practice for many hundreds of years (Previts et al. 2010; Sangster 2016), the increased need for standardized information, communicated via reliable and meaningful financial statements before and after the turn of the twentieth century, not only established financial accounting as an academic subject. This demand also motivated the normative-theoretical, qualitative-verbal research approach – apart from other supply-driven reasons that made statistically validated, quantitative and positivistic research less feasible in the early days of business administration in general. Especially, the Great Depression in the early 1930s increased the perception of financial accounting and reporting as a necessary and vital language of business that facilitates firm contract initiation and monitoring.

Whereas in the Anglo-American world, the emphasis has been more on (equity-) market efficiency (Zeff 2013), in other parts, for example in Continental Europe, financial information primarily served legal and debt-contracting purposes (e.g., Fülbier and Klein 2015). However, one parallel was obvious: Accounting research was viewed as an applied science. Academics actively cooperated with regulators, authorities, and jurisdictions as well as preparers; they did so by providing prescriptive conceptual underpinnings and interpretations to support the further development and application of financial accounting standards and techniques.

It is well-known that the ‘empirical turn’ in the U.S. at the end of the 1960s (esp. Benston 1967; Ball and Brown 1968; Beaver 1968) and the rise of Positive Accounting Theory in the 1970s (Watts and Zimmerman 1978, 1979, 1986, 1990), which was heavily contested (e.g., Tinker et al. 1982; Schreuder 1984; Whittington 1987; Sterling 1990; Boland and Gordon 1992; Mattessich 1995b), changed the situation. Since then, with increasing and nowadays arguably dominant importance, financial accounting research has been concerned with observable accounting behavior, its determinants and consequences, and the uncovering of cause-and-effect relationships. The research focus has moved away from standards and techniques, and the methodological approach changed to a pure positive science. Until today, data-based, quantitative, hypothesis-driven and positivistic research approaches have benefited from improved data availability and data processing capacity since the 1960s, advances in (finance) theory, and the econometrically sophisticated Ph.D. education programs primarily in the English-speaking world. Chua (1986) once referred to this as “mainstream accounting research” (see also Fig. 1), though this also suggests that other less dominant research streams exist, e.g., the more qualitative, conceptual, critical, interpretative or historical accounting research in the tradition of a social science with an interdisciplinary view that challenges the pure economic paradigm (Baker and Bettner 1997; Napier 2006).

In the German-speaking accounting research community, a specific “deductive-legalistic” research approach survived for at least another 20 years. Here, financial accounting was traditionally not designed primarily to assist (equity) capital markets by providing decision-useful information. Thus, there was also lower demand for empirical evidence about the value relevance and news content of accounting numbers from the perspective of shareholders. Due to the more tax-driven as well as profit-distribution focus of German GAAP, the research focus remained on standards and technical applications in practice (Busse von Colbe and Fülbier 2013; Fülbier and Gassen 2011). These approaches use stated accounting objectives to normatively derive prescriptive solutions to open accounting issues.^3^ They also conceptually provided ex-ante support and ex-post analyses of new accounting regulations during the second half of the twentieth century.^4^ Typical research approaches involve(d) the ‘critical assessment’ of accounting requirements, i.e., their benchmarking to overarching concepts and principles.^5^ Such deductive-legalistic, normative accounting research fell out of favor in North-American ‘top journals’^6^ long ago—to an extent where mainstream researchers discriminate these approaches as being “unscientific” (Jensen 1976, p. 12; Watts 1977, p. 54) or senior scholars apparently feel compelled to apologize for creating the impression of doing normative work.^7^

Albeit with some delay, the empirical and positivist turn towards “mainstream accounting research” also became established in the German-speaking community since the 1990s. The internationalization of accounting regulation towards the acceptance of IAS/IFRS in the European Union might have been a stimulating factor that increased the demand for effects analyses. Substantial pressures in the German university system to align accounting research with international approaches may also have contributed (Fülbier and Gassen 2011; Busse von Colbe and Fülbier 2013; Fülbier and Ruhnke 2022).

### Are we still making (the right kind of) progress?

We are clearly not the first to voice our unease about something being amiss in (“mainstream”) corporate reporting research. Senior colleagues such as Anthony Hopwood (e.g., 2007), Joel Demski (e.g. 2007, 2008, and as early as Demski et al. 1991), Stephen Zeff (e.g., 1989), Robert Kaplan (e.g., 1984, 2011, 2019), Sudipta Basu (2012), Wolfgang Ballwieser (2019), or Shiva Rajgopal (2020) have long been lamenting the state of accounting research, diagnosing a self-referential academic system with a preponderance of formalism and “rigor,” as well as a lack of innovation, intellectual diversity, passion for the accounting craft, and usefulness in practice.

However, a line of research has emerged that has contributed much to our understanding of accounting and corporate reporting. Becker et al. (2021), for example, document the positive impact of (some of the) IFRS-related research on academic as well as practitioners’ discourses, and on standard setting. Overall, since research targets different audiences, not everyone will find it equally relevant or understandable. Hence, it remains unclear whether non-academic constituents—for example standard setters with their need for relevant research (e.g., Beresford 1994; Beresford and Johnson 1995; Teixeira 2014)—have their needs addressed by the current research landscape (e.g., Fülbier et al. 2009; Becker et al. 2021, 194–200; Zeff 2021, with mixed evidence on this). Three anecdotes may illustrate our concern. First, Sir David Tweedie, the former IASB Chairman, once said in personal conversion to one of us that mainstream research has almost no impact on IFRS standard setting. Second, the German Federal Parliament (*Bundestag*) used to regularly consult business academics on accounting issues, but today legal scholars dominate parliamentary consultations. Third, the recent special section “International Accounting Policy Forum” in the 2022 issue of the journal *Accounting and Business Research* casts additional doubts on the usefulness of current accounting research for resolving longstanding standard-setting issues.

On the other hand, it seems as if the shifts described above, which took place about 50 years ago, have to some extent biased corporate reporting research, crowding non-mainstream approaches out of the outlets (in particular, academic journals) that largely determine academic careers in research-oriented universities. Although the turn to empirically more substantive research has generated many useful insights and initiated a meaningful new development at the time, it produced a negative side effect: a narrowing view of what is considered ‘good’ research. This bias relates to the choice of methods, theories, and substantive topics. In terms of methods, empirical-archival approaches have come to dominate, as many bibliographical studies have documented (e.g., Oler et al. 2010; Fülbier et al. 2014). Our theories are largely economics-based, with the ‘burden of proof’ seemingly being on researchers when evoking other theories to explain phenomena of interest.^8^

Regarding the substantive topics studied and the research questions posed, we feel that understanding has come to be viewed as more valuable than improving. For instance, the common framing of empirical work as testing the determinants (causes) and/or consequences (effects) of phenomena of interest betrays a positivist interest in eliciting the empirical regularities (treated as ‘laws of nature’) that characterize the role of corporate reporting in the economy. Much fewer top-tier publications appear to be dedicated to conceptual work intended to shape the practice of corporate reporting. It seems as if researchers find themselves in a vicious circle: Without statistically significant “evidence” on the effects of some reporting phenomenon (e.g., a certain sustainability reporting practice), they are hesitant to make suggestions regarding (improving) that practice – and the data needed to produce such evidence is typically unavailable unless the practice is implemented. It is also striking that some corporate reporting researchers primarily self-describe in terms of their preferred method-theory combinations (e.g., ‘I do experimental econ / game-theoretic modeling’), rather than the essential substantive questions they work on (e.g., ‘My research is dedicated to making corporate reporting an effective tool for nudging firms’ towards greater sustainability’). In consequence “manuscripts are the result of methods in search of questions, rather questions in search of methods” (Zeff 1983, p. 134).

The authors cited at the beginning of this section blame a biased academic incentive system, often described as ‘publish or perish’. Indeed, the described shifts and biases in research approaches arguably reflect researchers’ personal cost–benefit considerations. First, if researchers seek to minimize costs to themselves, they will favor research approaches for which the supply-related ‘factors of production’ (e.g., expertise, theories, methods, and data) are available to them at low cost (i.e., time and money). For example, Ball and Brown (2014), in a personal retrospective on their seminal 1968 paper, describe how an ‘explosion’ of empirical research in accounting and finance was catalyzed by the appearance of useful theories (i.e., efficient markets and the Capital Asset Pricing Model, CAPM), data (the Chicago Center for Research into Securities Prices, CRSP), computer processing power, and methods (the event study method developed concurrently with Fama et al. 1969). As a result, Ray Ball, Philip Brown and their Chicago colleagues, as well as William Beaver, who pursued similar research interests at the time, became widely perceived as role models. True, they themselves did pursue an approach that was risky at the time, and the ‘happy ending’ could not have been anticipated. For example, the work of Ball and Brown (1968) was first rejected as a finance paper by the established ‘The Accounting Review’ as the AAA’s core research outlet and top-tier mainstream journal at that time, before the authors submitted it to the newly founded ‘Journal of Accounting Research’ (e.g., Mattessich 1995b, p. 159). As a result of their out-of-the-box thinking, and probably also because there was an increasing demand for this type of research, PhD programs at major North-American PhD-granting institutions adapted their programs to accommodate the new research approaches, making the underlying theories, methods and data widely available to subsequent generations of researchers. These developments have dramatically lowered the relative costs of conducting certain types of research, and established a new mainstream period that continues to this day.

It is hence no surprise that new research streams have subsequently tended to spring up wherever a previously lacking ‘factor of production’ became available or less ‘expensive’. Such minimum factors often included data, but also empirical measures of phenomena of interest, or new methods. For example, newly available data sources tend to cause flurries of research activity, almost like rumors of gold attracting fortune seekers. In addition to CRSP and other commercial databases of accounting (e.g., Compustat), auditing (e.g., Audit Analytics), analysts (e.g., I/B/E/S) and capital market data, these include the public availability of EDGAR searches (e.g., Loughran and McDonald 2017), corporate earnings conference call transcripts (e.g., Mayew 2008), or Twitter messages scraped from the internet (e.g., Blankespoor et al. 2014), to mention just a few examples. Similarly, innovations in measuring key accounting concepts, such as earnings management using discretionary accruals (Jones 1991), earnings quality (see Dechow et al. 2010 for a review) or accounting conservatism (Basu 1997) have triggered enormous amounts of research. Finally, methods diffusing into accounting from other areas, such as automated textual analysis, have spawned entirely new streams of corporate reporting research (for a survey, see Loughran and McDonald 2016).^9^

Whereas newly available data, measures, or methods can critically advance research on important, long-standing questions, orienting one’s research agenda primarily by the availability of certain ‘factors of production’—instead of substantive issues—strikes us like an entrepreneur who cares little about what potential customers might want, but rather builds her business model around readily available resources. Much of corporate reporting research over the past years has relied heavily on settings characterized by low-cost availability of data in pre-structured, ready-to-use archives, often involving (U.S.) public firms and capital markets. These approaches fit well with the notion that, in capital-market-oriented accounting systems, decision-useful information is the vital base for efficient resource allocation (Zeff 2013), but may be of little relevance to other settings. Such “mainstream accounting research” (Chua 1986) was, and still is, clearly worthwhile for a better understanding of existing causal chains. However, where its comparative ‘competitiveness’ crowds out other settings and approaches, the progress of corporate reporting research on its essential questions is jeopardized. Investigating settings even where data is not easily accessible is important for a problem- rather than data- or method-driven research agenda, since settings in need of research are not necessarily those with easy data access. To address “problems of relevance” could be a helpful guideline for our research; this includes drawing more normative conclusions from empirical findings to aid decision makers.^10^ With or without data, we need creative research approaches, regardless of methodology (e.g., qualitative-conceptual, analytical, historical, or critical-interpretative in nature), that identify problems, create new insights, and develop innovative ideas and solutions in the field of corporate reporting.

Second, researchers—often trained as applied (business) economists—can be forgiven for behaving ‘rationally’ when it comes to the benefit side as well. Whereas the social benefits of corporate reporting research ultimately materialize in improved practices and policies that advance societal goals like resource allocation efficiency and distributive justice, individual researchers’ private benefits may only be indirectly linked to those societal impacts, if at all. In fact, many have observed a growing ‘research-practice gap,’ (e.g., Rutherford 2011; Federsel et al. 2022)—a disconnect between a detached research system that provides private benefits to researchers, and research that creates societal value by helping practitioners and policymakers solve problems. Therefore, the demand for the latter is unlikely to reach the researchers directly. At our conferences and in PhD programs, we discuss excessively about ‘how to get published,’ but rarely systematically expose ourselves and our students to the pressing questions of practice. One explanation for this gap is that research that benefits academics’ careers—in terms of top-tier publications, prestige, funding, income, and advancement—differs structurally from research that benefits society more broadly. In the words of Bob Kaplan (2019, p. 17), “scholars underinvest in research about practice innovations because such research is viewed as unpublishable in top-5 journals.”

For example, if innovation is primarily based on novel conceptions or critical reflections, neither of these two approaches is likely to clear the entry hurdles of the gatekeepers for highly-ranked journals. Also replications of important empirical insights in new settings can be difficult to publish there, resulting in perceived ‘replication crises,’ including, for example, in psychology (e.g., Maxwell et al. 2015) and, potentially, financial economics (Jensen et al. 2021). Related problems are on the one hand, that researchers exhaust themselves in the over-studying of the very same topics (McCarthy 2012); on the other hand, that they investigate increasingly marginal research questions that relate to phenomena, determinants and consequences of second- or third-order importance. To illustrate, where pioneering studies investigate relations of first-order importance (say, ‘Earnings quality and the cost of capital’), follow-up work will ‘play’ with different variations on this fundamental theme, including moderating factors (say, ‘Earnings quality and the cost of capital: the role of audit committees / manager overconfidence / …’) or alternative ways of measuring the variables of interest. Whereas such work can provide important complementary insights, research progressing in this incremental way is also at risk of addressing ever more marginal issues and failing to accumulate a solid body of knowledge on the core questions themselves. This risk goes hand in hand with a dearth of studies dealing with the further development of corporate reporting, although we need both, since normative implications and theoretical concepts and designs must be benchmarked against empirical evidence et vice versa.

It is not easy to explain why the social and private benefits of corporate reporting research would diverge in this way, especially in the long run. One (uncomfortable) possibility is lack of demand. Another way of expressing the above notion that researchers choose their projects to maximize private benefits, which overlap only marginally with social benefits, is to say that corporate reporting research strikes us as increasingly supply-driven rather than demand-driven. In Sellhorn (2020), one of us compares accounting researchers to virologists or epidemiologists, who have experienced unprecedented demand for their research during the Covid-19 pandemic. Clearly, virologists and accounting researchers are worlds apart. Perhaps our essential questions are less important than theirs? Where are the policymakers calling us about our latest insights, which they need for their decisions? Where are the large research grants for our field?^11^ If researchers do not receive clear signals of societal demand for their research, perhaps working in a supply-driven and self-interested way is a rational response—in particular for junior colleagues who need the publications to establish themselves. These emerging scholars can be forgiven for being too risk-averse to venture off-mainstream—also considering that many institutions cannot make credible commitments to permanently employ them if they perform well. Thus, if corporate reporting research is to change, corresponding shifts in these framework conditions seem necessary.^12^

## Why change is needed

In this section, we argue that corporate reporting research needs to change to address new, increasingly difficult problems as well as new facets of long-standing issues. This requires, in particular, a shift from method-driven to problem-driven research.

### Refocusing on ‘wicked’ societal problems

Perhaps corporate reporting researchers need to become bolder and realize that they, albeit not by themselves, have the capability of helping address many of the ‘wicked’ problems we are facing today.^13^ Without any claim to completeness—and certainly without implying that we have the answers—we restrict ourselves to discussing just one of these issues: environmental and social degradation, which is closely linked to climate change—and which some refer to as environmental justice. Few people now doubt anymore that anthropogenic climate change is real, and that urgent action is needed. As academic accountants, one of the key concepts around which our research and teaching revolve is information. Information is also increasingly at the core of the environmental policy debate. We are seeing rapid developments in the landscape of global corporate financial and sustainability reporting—with environmental disclosures being positioned as one of the potential solutions to climate change and related environmental and social problems. The IFRS Foundation and the European Commission are very much at the center of these tectonic shifts.^14^

However, it is still unclear what it takes for such information to fulfil that promise. Is measurement the key, perhaps going as far as monetizing firms’ environmental and social externalities for inclusion in financial accounts (‘impact accounting’ in Table 1)? For a century, (income) measurement has been at the center of accounting thought, and accounting researchers can be said to have a competitive advantage when it comes to measurement.^15^ In the light of the growing research-practice gap discussed above, which some argue threatens the very relevance of our discipline to real-world problems, the current planetary-level environmental and social challenges provide an opportunity for accounting academia to prove that we do matter.

### Changing parameters of corporate reporting practice

As indicated before, we like to believe that corporate reporting is at the beginning of a new dynamic age, shaped by disruptive challenges to planet and society. The core conceptual premise remains the same: information asymmetries and conflicts of interest exist between firm insiders and outsiders, as well as among outsiders, which need to be reduced in order to allow contracting and monitoring in formal or informal principal-agent relationships. However, several parameters of this conception are currently changing.

#### Information demand

New purposes: Many of the environmental problems we face (most notably, climate change) are market failures in the form of environmental externalities. One of their causes is information asymmetry: firms’ actions degrading environmental resources without stakeholders being sufficiently aware or empowered to hold managers accountable. Public disclosure is one way of overcoming this problem. In fact, mandatory disclosure is increasingly being used as a regulatory tool that might be less intrusive than traditional command-and-control regulation or financial incentives set via subsidies, taxes, or price regulation. Disclosure regulations like the mandatory public display of hygiene inspection results in restaurants (Jin and Leslie 2003), nutrition labelling by food producers, or the disclosure of mortality rates by hospitals (for a summary, see Fung, Graham, and Weil 2007, Hombach and Sellhorn 2019) are targeted towards fostering specific policy objectives by ‘nudging’ economic agents towards (or away from) certain actions, and hence have been ‘targeted transparency’ (Weil et al. 2013). We need research into what makes public disclosure *effective* (in terms of successfully ‘nudging’ firms towards more sustainable actions) as well as *efficient* (in terms of being less net costly than other potential remedies, such as a Pigouvian tax) as a regulatory tool.

New users: The long time prevailing shareholder perspective is changing into a stakeholder perspective. The focus on (equity-) capital market participants especially in international, capital-market oriented accounting is expanding—reflecting a shift from shareholder- to stakeholder-centric theories and conceptions of the firm.^16^ While financial capital has been the dominant bottleneck for economic growth over the last 100 years, this focus has shifted. In a time of lower interest rates as a result of expansive monetary policies by central banks, financial capital has become to a lesser extent a scarce resource. Non-investor stakeholders are moving more into focus, with skilled labor becoming scarce, especially in many Western democracies with demographic problems, and therefore gaining more attention. Simultaneously, the values and needs of these employees are changing, and especially younger generations seem to insist more on equal and non-discriminatory participation, minimum social standards, and environmental protection (e.g., Rodrigo and Arenas 2008; Glavas 2012; Casey, and Sieber 2016).

Corresponding co-responsibilities arise for the corporate sector, reinforced by large and increasingly vocal institutional investors and asset owners (see BlackRock as an example; e.g., Fink 2020) as well as social movements (e.g., Fridays for Future, Extinction Rebellion, or 350.org), which raise the strategic question of corporate purpose (Basu 1999; Mayer 2018). Greater transparency—not only about firms’ financial risks and opportunities, but also about their social and environmental impacts, corporate governance issues—is a possible vehicle for effecting change.

Another two stakeholder groups are receiving more attention in financial accounting than before: customers and suppliers. Their interests are no longer limited to product quality and the financial stability of the contract partner. Instead, there is an increasing demand for information about the environmental dimension of the production process, and about compliance with minimum social and ecological standards throughout the supply chain (e.g., Delmas and Montiel 2009; Caniels et al. 2013), for which firms are increasingly held accountable (e.g. *Lieferkettensorgfaltspflichtengesetz* 2021 in Germany; proposal for an EU Corporate Sustainability Due Diligence Directive 2022). Even the traditional users of corporate reporting, the capital providers, are expanding their information needs in a similar direction, to ensure that their investments meet the increasing social and environmental minimum requirements (e.g., Migliorelli and Dessertine 2019; Schoenmaker and Schramade 2019; Fink 2020).

Changing information needs are also reflected in two other groups of users: The interested public and governments. Whereas the former has long been ignored by standard-setters and much of academic research, the latter has been considered mostly as a fiscal authority, or in its regulatory and administrative roles (e.g., price regulation or accounting enforcement). Both views seem narrow. By definition, the interested public is interested because they suffer (or benefit) from the externalities that firms cause. On behalf of the affected public and society as a whole that develops these new information needs, the state acts as a guardian of their interests, with elections creating the respective incentives for politicians. All state instruments of power, especially legislation and administration, are currently aligning with these objectives.

These trends help explain the demand for more comprehensive corporate information reported to outside stakeholders. In consequence, a lot of open (research) questions come into focus that deal, amongst others, with the different and possibly divergent stakeholder preferences, the identification of their information needs as well as the effective and efficient standardization of corporate reporting instruments and their integration into management accounting systems.

More uncertainty: Changing users and their information needs triggered by societal and environmental developments are material aspects of the current situation. But other parameters are also shifting. The long-term stability of contractual relationships as well as institutional settings seems to be eroding. Firms are dealing with higher volatility and shorter-term pressures in many of their contractual relationships—including due to higher transparency and faster feedback loops via the internet and social media. Moreover, political and economic crises such as Covid-19, supply-chain disruptions, and the Russian attack on Ukraine seem to emerge more and more frequently, forcing all affected actors to adapt to greater uncertainty in their planning. Thus, a carefully coordinated strategy, a sustainable purpose, and the resilient and agile management of contractual relationships, by providing timely and decision-useful information that is not only tailored to investors are becoming increasingly important for firms. Research might help by assisting the development of more timely and more comprehensive corporate reporting instruments that provide on the one hand a better understanding of estimates, forecasts, risks and opportunities, and, on the other, more individualized and stakeholder-specific access to useful information.

#### Information supply

Technical aspects of information production and dissemination are changing due to progress in digitalization and automatization. We are currently experiencing dramatic and, for some industries and business models, disruptive changes in information technology. The exponential growth in data and its availability, the ever-improving information processing capacities and the still not fully foreseeable opportunities of artificial intelligence affect corporate accounting and reporting. At its core, accounting is about information—information that can now be generated as well as retrieved much more timely and comprehensively at ever lower cost, and perhaps with greater accuracy. Most likely, these trends, too, will change the entire infrastructure of corporate accounting, reporting and auditing—and will raise new research questions and opportunities.

### Resultant need for change in corporate reporting research priorities

If researchers express their values, worldviews and priorities through the research questions they choose to address and the research approaches they select (e.g., Chua 1986), and if research presented at leading conferences (e.g., Fülbier et al. 2014), by leading scholars (e.g., Fülbier and Weller 2011) and published in leading journals (e.g., Oler et al. 2010) is any indication of the research being conducted in an academic field, corporate reporting researchers in the last 50 years have largely cared about the efficient functioning of Western capital markets and, to a lesser extent, about holding the managers of public corporations accountable for the shareholder value created by their firms. In the process, we have learned much about information processing and firms’ information environments (e.g., reviews by Kothari 2001; Beyer et al. 2010; Blankespoor et al. 2020), accounting choice and earnings management (Fields et al. 2001), the role of financial intermediaries such as analysts (e.g., Brown et al. 2016, 2015), the determinants and consequences of accounting standards and behavior (e.g., Zeff 1978; Hagerman and Zmijewski 1979; Holthausen and Leftwich 1983; Ewert and Wagenhofer 2005; Ernstberger et al. 2012; Leuz and Wysocki 2016to name just a few in this immense literature), as well as other, related areas. These studies have largely relied on economic theories and empirical-archival methods or, less often, analytical models.

As indicated earlier, these mainstream approaches with their focus on ex-post observable data appear to be faced with diminishing marginal utility in the light of the changes and challenges outlined above, as the parameters of financial as well as non-financial reporting as the language of business are changing dramatically. We now again see reasons that make a refocusing of research priorities and resources opportune—comparable to the beginnings of financial accounting regulation approximately 100 years ago, or the factors that brought about the ‘empirical revolution’ about 50 years ago. We face the dramatic challenges and opportunities of digitalization, automation, social inequalities and—maybe the major task of the twenty-first century—environmental decline. Along with the natural sciences, the humanities, as well as the engineering, medical and formal sciences, business economics as part of the social sciences, and corporate reporting in particular, should contribute to answering these new questions.

To us, this implies a visionary reinvention of the language of business. ‘Looking back’ at the status quo using research approaches that seek primarily to understand ‘what is’ remains useful, but might lose its dominant role^17^—at least, if we do not want to leave the further development of financial accounting (regulation) exclusively to policymakers and practitioners. ‘Looking ahead’ with the courage to propose scientifically sound, prescriptive solutions—in the tradition of design science and design regulation research (e.g., Hevner et al. 2004; Fülbier and Seitz 2021)—seems also important for improving the situation and offering solutions proactively. After all, research that limits itself to the ex-post assessment of existing practices and policy interventions has limited scope for contributing ex-ante insights that help develop effective practices and policies in the first place (e.g., Fülbier et al. 2009). In the long term, those limits will most likely have an impact on the academic market as well. With multiple crises wreaking havoc on academic institutions’ and government’s finances, the current, more method-driven research may end up facing drying-up funds if there is no ‘real impact’ on society (Sellhorn 2020). Similar concerns are being voiced in other social sciences, including psychology (e.g., Sternberg 2007). The funding issue seems crucial for getting researchers and universities to focus on socially relevant problems, at least in the long term. To a certain extent, the beginnings of this development are already visible today when we observe the increasing efforts of universities to build up teaching and research capacities in the field of sustainability. Sooner or later, there will be a reorientation of corporate reporting research, either through researchers’ own awareness, or due to funding constraints. In such research, a community of scholars will (hopefully) pursue whatever approaches they each practice best in the common, often interdisciplinary search for solutions—with whatever methodological and epistemological orientation, theories, data sources, econometric methods, and institutional settings turn out to be useful (Feyerabend’s 1993 ‘anything goes’).

## How to get there

In this chapter, we present some of the areas where we consider more *issues-driven* and *applied* research needed—research designed with the objective of contributing to important societal problems in mind. In order to base our assessments not only on our own personal judgment, we collected input from about two dozen senior colleagues and high-level practitioners in the German speaking area.^18^ We realized that most of the received opinions converged in the direction outlined below—our original assessments were quite in line with this, and we feel, thus, more comfortable in expressing our thoughts. Since diagnosing problems and demanding more research on this or that are important but not enough, we also try, in all modesty, to propose possible paths towards concrete potential solutions. Since this chapter touches upon many broad and deep streams of literature, we may be excused for citing only sparingly and without any claim to representativeness or even completeness. Also, we adopt primarily a decision usefulness perspective, although we are well aware (and address it sporadically) that financial accounting in particular has evolved to serve other purposes, especially contracting, as well.

### Improving financial accounting (standards)

Financial accounting, in the form of standardized financial statements and notes, remains important for information purposes. However, there are long-standing accounting issues that seem to remain unresolved, at least by standard setters. Hombach and Sellhorn (2022, p. 543) consider an accounting issue resolved to the “degree to which the established accounting solution successfully reduces mapping uncertainty and undesired consequences”, where mapping uncertainty arises “typically on the part of preparers (and, to some extent, auditors), where it is not obvious how an economic transaction or event should be mapped into an entity’s financial reports.”^19^ Unresolved accounting issues often (re-)appear repeatedly during standard-setters’ agenda consultations and are the subject of persistent debate in corporate reporting literature. In this section, we selectively discuss the following issues, which we consider unresolved and likely to benefit from further research: (a) role of (traditional) financial statements, (b) intangibles, (c) business combinations, and (d) pollutant pricing mechanisms.^20^

Role and content of (traditional) financial statements: We still need a better conceptual understanding about the role of financial-statement-based content in reporting, firm contracting and management. This understanding is essential to advance and improve standard setting. Financial accounting figures and ratios are used for valuation purposes but also firm contracting, for example in debt covenants, as important indicators of financial position and performance. Trade-offs exist and are sometimes addressed as “unintended consequences” (e.g., Brüggemann et al. 2013). Accounting changes for better investor information trigger, for example, contractual or legal consequences, as long as ‘rolling GAAP’ is used in these contracts.^21^ Key figures and ratios also play a similar role in value-based management and executive compensation. Research has the potential to answer open questions for public and private firms in different institutional settings, for example, about the acceptance of financial information for reporting, contracting and management purposes, about their conceptual role, acceptance and effects—also with regard to historical cost as well as fair values (and their combined use)—and about the challenge of integrating non-financial indicators (see below). Another aspect of the very same problem is the accounting entity: Currently, financial statements in corporate reporting especially on capital markets abstract from the legal entity and focus on the economic entity, the group. However, from a broader stakeholder perspective, assets shifting within the group are possible with exploitation potential for some contract partners of some legal entities. Thus, a more intelligent interplay of group and legal entity accounting and reporting seems appropriate to capture these trade-offs.

Against this background, we still do not know exactly what the content of the (traditional) financial statements should be. It is still an open question whether it is a reasonable objective (if only for investors) to reflect market capitalization in traditional financial statements. This is not only a question of normative research. Positive empirical research can also contribute. Do users find it important that net assets should reflect market capitalization? Research might help answer this question with regard to different stakeholder needs and institutional settings. An additional issue is the integration of forward-looking information. Although forward-looking information about the firm’s prospects does enter into the recognition and measurement of many classes of assets (e.g., receivables, intangible assets) and liabilities (e.g., provisions), financial accounting has been characterized as mostly backward-looking, whereas projects and forecasts are largely found in additional disclosures, e.g., within the German management report^22^ or the U.S. Management’s Discussion and Analysis (MD&A). To the extent that unpredictable disruptions and crises render the time series of past events and transactions (and their accounting realizations) bad predictors of future events and transactions, the demand for new approaches to providing forward-looking information is increasing. One key feature of such disclosures is that they explicate the uncertainty inherent in the predictions, for example based on scenario analyses to assess the resilience of the entity. Since sophisticated scenario analysis and other forms of explicating uncertainty are currently relatively rare in corporate reporting, researchers can study how such techniques can most effectively help reduce information asymmetries and perceived uncertainty while at the same time providing a mechanism for credible signaling.

Intangibles: For a long time, we have been trying to better integrate intangibles into the financial statements—especially the internally generated intangibles. To solve this eternal challenge of financial accounting is probably more important today than ever before to better capture the new value drivers in our knowledge- and technology-driven societies, for example usable data, data access options, own digital platforms and algorithms. Prior empirical research provides evidence that users of financial statements are asking for (useful) information in this regard (e.g., Lev 2001, 2019; Zambon and Marzo 2007; Wyatt 2008). Additional empirical research can deepen our understanding how this need for information varies across different stakeholder groups, different categories of intangibles, and different institutional settings. Moreover, conceptual research efforts in the tradition of “design regulation research” (Fülbier and Seitz 2021) are necessary to determine to what extent the recognition, measurement and disclosure of intangible resources need to change from current standards. Consequently, the IASB and EFRAG discuss a project on intangible assets with a comprehensive review of all aspects of IAS 38 to better reflect the increasing importance of those assets in accounting and corporate reporting (e.g., IASB 2022, EFRAG 2021). Richard Barker and Stephen Penman argue that the critical issue is not the intangible or tangible nature of a resource, but rather the uncertainty associated with its expected future cash flows. They argue that “conditions of uncertainty render both the balance sheet and the income statement ‘incomplete,’ yet complementary, with respect to the IASB’s objective of providing decision-useful information to investors” and “propose an income-statement approach to financial reporting that extends (and complements) the balance-sheet approach that is embedded already in the Framework, a ‘mixed’ approach” that conveys “information about uncertainty” (Barker et al. 2020, p. 324; see also Barker et al. 2021).

Further research seems required to understand if we have a knowledge deficit or an implementation deficit here. Against the background of many past decades of discussions about intangibles (as just one example, refer to Lev and Gu 2016, also Zambon et al. 2020), it seems interesting to ask why standard setters seem to struggle with reforming the accounting for intangibles, what the societal harms of current rules are, as well as whether and how this stalemate may be resolved (Lev 2019).

In line with prior considerations about the role of (traditional) financial statements, the possible inability of balance sheets to capture more comprehensively the firm’s market capitalization might foster a complementary solution, a kind of intangible capital statement. Prescriptive research and also practice have already suggested several alternatives in this regard, without resounding success, not to mention a standard for a new financial statement. Research might illuminate the reasons for inertia so far, it might additionally provide more guidance about the framework and possible content—financial or non-financial in nature. Suggested categories of intangibles (e.g. the seven intangible capitals by WGARIA 2005; partly based on Edvinsson and Malone 1997) could benefit from newer interpretations in terms of social capital, which might be part of human capital characterized by anti-discriminatory, equal, family-friendly and flexible working conditions, important for the new generations of employees or other aspects that reach, for example, into the area of process capital with compliance and governance conditions within a firm’s organization. The transition to the major topic of sustainability reporting seems fluid at this point.

Business combinations: To improve goodwill accounting is a long-lasting challenge that touches not only the still controversial debate about the subsequent measurement of the acquired goodwill, where research is highly appreciated to move forward on this path. Very much in line with the prior point, the recognition of intangibles (and other assets and liabilities) in M&A transactions is again an issue, from which goodwill results in consequence, either as full or partial goodwill. In their recent review of the related literature, Amel-Zadeh et al. (2021) conclude that goodwill amounts, on average, are associated with the underlying economics of the combining firms but are also shaped by managerial incentives and institutional context. However, empirical-archival research alone is insufficient to assess whether current requirements provide for an optimal degree of discretion. Calling for more research in this area, Amel-Zadeh et al. (2021) advocate reproduction studies to test the generalizability of existing findings across contexts, and encourage standard setters to initiate quasi-experiments to generate causal evidence and to render policymaking more accountable. They further suggest that researchers make more use of behavioral theories and non-archival methods to elucidate the motives and interactions of decision-makers in goodwill accounting. Such research could aid the normative development of better conceptual guidelines to increase transparency about measurement parameters and additional assumptions. One particularly pertinent area in which greater transparency is needed relates to ex-ante forecasting and ex-post documenting the performance of business combinations by preparers.

In this context, the IASB’s current discussion paper “Business Combinations—Disclosures, Goodwill and Impairment” (IASB 2020) proposes, among other things, improved disclosures about the subsequent performance of acquisitions, stating “that companies typically do not provide enough information to help investors understand the subsequent performance of an acquisition. Investors cannot assess whether management’s objectives for the acquisition are being met—for example, whether the synergies management expect from an acquisition are being realised” (para. 2.4). As Sellhorn (2021) points out, practitioners often argue (and understandably so) that the expected synergies underlying purchased goodwill can hardly be tracked over the long term; through integration and restructuring, the acquired goodwill is inextricably mixed with other values. Apparently, even after a short time subsequent to an acquisition, many companies can no longer tell whether a deal has actually generated the expected values. Research of any kind may help firms and standard setters devise and implement approaches to measuring and documenting the subsequent performance of acquisitions, to enhance transparency and accountability. Such approaches may also help resolve another long-standing conceptual issue: “Is goodwill an asset?”^23^ After all, where an acquirer cannot convincingly justify, in terms of expected future benefits, the purchase price paid in an acquisition, nor track the subsequent arrival of such benefits, the asset nature of the corresponding accrual ‘goodwill’ is in severe doubt (Sellhorn 2021).

Pollutant pricing mechanisms: As summarized in Hombach and Sellhorn (2022), accounting issues related to pollutant pricing mechanisms such as carbon emissions trading schemes pertain to the resources and obligations arising from such schemes, especially where emission allowances are received free of charge (e.g., Bebbington and Larrinaga 2008). Since the IASB’s removal of its IFRIC 3 *Emission Rights* in 2005, over concerns about accounting mismatches, this issues in unregulated under IFRS, triggering calls for a standard-setting solution (e.g., Elfrink and Ellison 2009). One approach to providing evidence-based normative guidance on such issues is exemplified by Ertimur et al. (2020) who use publicly available data to simulate different conceptually derived possible accounting treatments, and then compare the value relevance of accounting summary measures under each method.

### More targeted and efficient standard setting

Many of the challenges in corporate reporting go along with standard-setting implications since policymakers (and forces influencing them) are often likely to be drivers or at least catalysts of these developments. Thus, the standard-setting process and environment also deserve more attention in research in order to better understand and to improve the language of business.

Information needs of diverse stakeholder groups: If users do not retrieve corporate information based on their individual preferences (see this point discussed above) standard setters have to provide for standardized information that corresponds to the information demand of at least the aggregated major user groups. The better research is able to identify, disentangle and understand the group-specific information needs in different settings, the more suitable corporate reporting devices can be designed. This kind of research contributes to a more consistent means-end logic in corporate reporting standard setting.

Balanced standard-setting participation of stakeholder groups: Better understanding the information needs of stakeholders goes along with more balanced participation and articulation of these groups in the standard-setting process. Prior research documents that users rarely participate, that preparers and auditors have an overall disproportionate influence, and other political forces (e.g., Sutton 1984; Gaa 1988; Saemann 1999; McLeay et al. 2000; Zeff 2002; Becker et al. 2021, 151–182). This imbalance creates a legitimacy problem for standard setters espousing decision usefulness as a core objective, as well as an information problem, to the extent that users’ information needs cannot be observed. In such cases, standard setters as well as preparers can do little but “construct” users information needs based on their own assumptions and private interests (Young 2006; Oberwallner et al. 2021). In order to get closer to understanding users’ needs, research might assist in solving the problem that the costs of accounting standards (incurred mostly by preparers) are immediate and easily measured, whereas the benefits to users, and hence also to preparers can appear distant and elusive. It seems worth discussing possible solutions, for example to involve researchers more intensively than before in standard-setting as advocates of users, due to their more neutral role, which is hopefully driven by research findings of all kinds, either from conceptual considerations or empirical evidence, rather than self-interest.

Cost–benefit analysis of reporting standards and regulations: Every regulation in a liberal society with a market economy requires ex-ante justification. To accomplish this task in the field of corporate reporting, normative regulatory theory with reference, for example, to welfare- or microeconomics is useful for conducting convincing cost–benefit analyses (Fülbier et al. 2009; Schipper 2010; with Feldhoff 1992; Fülbier 1998 as examples). These approaches describe an important interface between legal and economic research in the tradition of Posner (2014) and others who examine the economic effects of legal rules. Non-economic analyses from the natural or social sciences, also with reference to higher-level social values, for example in the context of sustainability (e.g., DesJardins 1998; Poff 2010; Steffen et al. 2015), can enrich these justifications in a more interdisciplinary way.

Evidence-informed standard setting and policymaking require additional ex-post assessment against ex-ante objectives (Teixeira 2014; Fülbier et al. 2009). Hence, opportunities for standard setters and researchers to collaborate could include committing ex ante to a specific ex-post assessment in the context of Post-Implementation Reviews (PIRs; e.g., Ewert and Wagenhofer 2012). This would imply specifying observable outcomes that reflect a standard’s objective, for example, a change in the number of consolidated subsidiaries around the adoption of IFRS 10 (e.g., Bedford et al. 2022) or in the number of reported operating segments upon the introduction of IFRS 8 (e.g., Moldovan 2014).^24^ Data availability could be secured by mandating that firms provide certain data in a way that is easily accessed by researchers (Leuz 2018). To generate even more direct causal evidence on the effects of new standards, standard setters and researchers together could devise formal field experiments by which some randomly selected treatment group of firms adopts (pilots) a new requirement before other firms (the control group) do. This suggestion may seem an academic’s ivory tower dream, but as Leuz (2018) documents, has several real-life precedents: “A good example is the Regulation SHO pilot programme that the SEC did on short sale restrictions (e.g., Li and Zhang 2015). Another example is the FINRA tick size pilot programme. I would encourage regulators to perform such pilot studies (with randomisation) more often” (p. 600).

Commitments to transparent evidence-based standard setting, including systematic ex-post assessments, may also ease constituents’ concerns about regulatory overreach, since ineffective or inefficient requirements would be weeded out, with only those being retained that ‘survive’ ex-post cost–benefit analysis. One way to achieve this by giving new requirements an ‘expiry date’ whereby they are rescinded unless shown to be effective and efficient during ex-post assessment. For example, the current situation in the evolving field of mandatory sustainability and ESG reporting is characterized by multiple and massive efforts to standardize and regulate additional reporting requirements. Research might help to critically assess these efforts ex ante as well as ex post. The faster these regulations will be developed and deployed, the more important is ex-post assessment—the analysis of whether the regulation has successfully achieved the regulation objective (effectiveness) under cost–benefit considerations (efficiency). Moreover, if massive new non-financial information will be generated due to new sustainability requirements, some of which serve changing stakeholder information needs, the more ‘traditional’ reporting requirements should be reviewed and, if necessary, reduced or eliminated.

Last, but not least, and linked to the previous point, we need more evidence on and (conceptual/theoretical) understanding of the alleged problem of ‘information overload.’ In times of digitalization and big data, is it really true that more information causes more costs, for preparers including proprietary costs (Verrecchia 1983), and especially to users? What constitutes the information overload? Research might assist here in better understanding the information production by preparers as well as its acquisition and processing by users. The more traditional understanding that users read and digest all the given information might be challenged by a different processing model, where users, using intelligent search strategies, access only certain selected information and expect a great(er) amount of detail there.

Admittedly, many of the above suggestions are not new (e.g., Fülbier et al. 2009; Ewert and Wagenhofer 2012; Leuz 2018). However, the question remains why these insights have triggered so few changes in real-life standard setting. Economic theory typically suspects that the issue lies with the incentives of the actors involved. Clearly, corporate reporting standard setting and policymaking are shaped by constituent lobbying—as indicated above. Therefore, the challenge for academic research is not only to provide conceptual and empirical insights to standard setters and policymakers, but also conduct further research into the factors that promote or hinder the ‘translation’ of such insights into actual standards and policies. A rather new strand of literature also highlights the political influence of special interests and ideology on reporting regulation (e.g., Bischof et al. 2020; Becker et al. 2021, 151–182). Here, too, more evidence is needed, especially in the rather new context of sustainability reporting.

### Making targeted transparency regulation effective and efficient

Legitimacy of targeted transparency: Corporate reporting is more or less globally regulated and standardized at the national or supranational levels – primarily with a view towards allocation efficiency and capital provider protection. However, the current discussion about sustainability reporting might introduce a different logic: Targeted transparency regulation. Introduced above, targeted transparency regulation uses reporting requirements to promote policy objectives, and as such represents one among several types of policy interventions that vary in the extent to which they interfere with the market mechanism.^25^ Here, corporate reporting becomes more political in nature: The regulator has a ‘steering function’ in mind, driven by a political agenda. Guiding corporate decisions in a particular direction seems more important than providing (neutral) information to support decision-making in whatever direction. Is targeted transparency regulation a legitimate regulatory instrument for fostering policy objectives? If research will contribute in this regard, more investment seems necessary to better understand the role of corporate reporting as a means to an end. Is this really a new challenge for research or quite similar to current and prior times where information flows to capital markets have been justified (also by researchers) with reference to the market efficiency doctrine? This includes an economic analysis of alternative instruments to foster the corporate incentives for a more ESG-compliant behavior. At the meta level, an additional political and philosophy-of-science debate seems inevitable: To what extent do research and researchers want to serve politics and politicians (one could also say “society”) – either by developing instruments for a given political agenda (i.e., conditionally normative; Mattessich 1995a, 1995b) or by proposing their own normative agendas (purely normative)?^26^ Although the sustainability goals may seem unquestionable to many (and, to the extent they are the subject of international accords like the Paris Agreement, in fact *are* legally binding), this might not be true for sub-goals or entirely different objectives, including ideology-driven ones. However, this discussion is linked again to the one about value judgments in science, which has not been settled even after more than 100 years (Fülbier and Weller 2008).

Causal mechanism of targeted transparency: If targeted transparency regulation is viewed as legitimate, it is helpful to conduct *ex-post* studies of targeted transparency regulation already implemented (e.g., Christensen et al. 2017; for a review, refer to Hombach and Sellhorn 2019). There is also a need for more *ex-ante* research that helps with the normative design of effective and efficient policy interventions (Fülbier et al. 2009). Such research needs to unpack the causal chain of mandatory public disclosure, stakeholder actions, and firms’ adaptive responses that underlies the ‘targeted transparency action cycle’ (Fung et al. 2007; Weil et al. 2013). As discussed in Hombach and Sellhorn (2019), the effectiveness of such requirements hinges on stakeholder pressure facilitated by previously private information, for example about a firm’s environmental externalities, forced into the open and exposed to public scrutiny. This causal mechanism consists of several links, all of which can be characterized by individuals and their responses to new information. Some expect (or hope) that increased transparency can be part of the solution to some of the ESG issues the world is facing. Research of all kinds needs to further ‘unpack’ these links to provide insights into ways of making targeted transparency more effective and efficient towards this end.

Establishing impact accounting: We are in the middle of a societal debate about how best to achieve sustainable development (hereinafter exemplified by net-zero GHG emissions, or the Paris Agreement’s 1.5° goal). Climate change and other environmental and social issues are generally understood as representing market failures that arise from the failure of corporate costs and profits as well as market prices to reflect the externalities caused by firms, leading to overconsumption of natural resources. Corporate reporting needs to evolve towards capturing the ecological externalities that business entities cause. Reflecting these externalities in the familiar ‘language of business’ is likely to contribute to their internalization. Among the four largely distinct information systems that comprise corporate reporting (financial accounting, financial disclosure, impact accounting, and impact disclosure; see Table 1) sustainability-related financial disclosures (e.g., according to evolving IFRS Sustainability Disclosure Standards) and sustainability-related impact disclosures (e.g., according to evolving European Sustainability Reporting Standards, with an impact materiality focus) already provide hopefully valuable information that empowers stakeholders to hold firms accountable. Research seems important here, given the many unanswered questions. How do we solve the core problem of standardized measurement of many completely different reporting dimensions? How can we incentivize firms to provide a true and fair view of their business activities—including their impact on, and exposure to, sustainability-related matters (double materiality)—without overburdening them, thus weakening their international competitiveness, but rather offer solutions with a view to readjusting business models and opening up new markets? How do we achieve a meaningful balance between relevance and verifiability in view of sometimes very long observation periods, as well as numerous complex interdependencies and trade-offs between the “E”, “S,” and “G” areas? These highly relevant questions refer to solid but not exclusively economic analyses on theoretical or empirical grounds. They also increase the need for more prescriptive studies providing novel ideas, guidelines and recommendations in the tradition of design science and design regulation research (e.g., Hevner et al. 2004; Fülbier and Seitz 2021), based on conceptual work or, if related to existing experiences, on empirical evidence.

We might also benefit from previous research on financial reporting when it comes to similar questions in the non-financial sphere. With the many regulatory efforts around the world in sustainability reporting, the path to one globally accepted language in this area, comparable to IFRS, might benefit from the multi-faceted research on IFRS that has examined the costs and benefits of IFRS as a single set of global reporting standards, the market consequences of IFRS adoption (Brüggemann et al. 2013), the global practices of IFRS reporting, and the political economy of IFRS (Becker et al. 2021).

An even greater and more direct impact can be expected from novel approaches to impact accounting, which translate firms’ ecological and social externalities into the language of financial accounting: monetary units. For example, Quattrone (2021) and Barker and Mayer (2021) propose in their conceptual studies to extend the traditional financial accounting income statement by adding a monetary estimate of the firm’s environmental externalities. In contrast, current sustainability requirements proposed by the ISSB and EFRAG stop short of requiring the monetization of corporate externalities and their integration into financial reports. With a similar motivation, but pursuing a different approach, Sellhorn and Wagner’s (2022b) ‘Paris-aligned IFRS financial statements’ concept seeks to simulate what firms’ IFRS accounts would look like if these financial statements had been prepared under assumptions in line with a world firmly entrenched on a transition pathway towards the Paris 1.5 °C goal.

Determinants, consequences, opportunities, and limitations of transparency: Although countless studies have contributed to our understanding of transparency, the complexity of this topic is such that more research will be necessary to reach a better understanding. These efforts should consider the full range of stakeholders, contractual relations (including in private firm settings), different institutional settings, the special features of non-financial reporting, the different roles of regulated, mandatory and voluntary transparency, real effects, cost–benefit considerations, the impact of big data and modern information technology, and other factors. If the shareholder focus of corporate reporting will change into a broader stakeholder perspective, research of any kind has to contribute further knowledge about the different information needs of users and their information processing capabilities in different settings, as well as the stakeholder-driven real effects. This understanding seems necessary to design and improve a stakeholder-oriented corporate reporting. In the German academic context, this research agenda is pursued by the DFG-funded Collaborative Research Center TRR 266 *Accounting for Transparency*.^27^

### Harnessing digital transformation

At its core, corporate reporting is about information—information that can now be generated as well as retrieved much more timely and comprehensively, and perhaps with greater accuracy. Most likely, this will change the entire accounting, reporting and auditing infrastructure.

Opportunities and boundaries of big-data-based corporate reporting: We need more research of all kinds to understand the impact of big data in an information technology environment on corporate reporting. Will the (real or perceived) problem of information overload (see above) increase or decrease? What about sensitive information, internal planning data, the level of aggregation, the cost to collect and process information to preparers and users? What about the opportunities and risks of artificial intelligence applied to accessing and analyzing corporate information from public sources and the internet? What are the technical challenges, what are the economic consequences? How to combine the power of digital information technologies with human expertise and judgment to address ‘wicked problems’ or ethical-moral dilemmas? The range of research questions seems endless. However, the opportunities to develop new and better reporting instruments seem also fascinating. Two examples follow where research can also contribute.

*Designing financial and non-financial reporting instruments and channels with instant user access to information:* The traditional need to wait for annual or quarterly information may transform to timelier and individual solutions. Practice, hopefully with researchers’ assistance, might develop a reporting environment that provides information about a self-selected reporting period at a self-selected point in time, virtually at the push of a button (Pellens et al. 1998, Gassen 2000; Pellens 2007). These new information systems come along with formidable challenges that need to be solved: To ensure retrieval at any time, we need design science efforts (Hevner et al. 2004) to develop continuous, real-time accounting with continuous data collection, closing entries, continuous earnings management decisions as well as continuous, real-time auditing efforts. Research of any kind is also necessary to investigate challenging follow-up questions related to the (proprietary) costs and benefits to preparers of such ‘extreme’ transparency.

*Designing financial and non-financial reporting instruments and channels that better reflect individual user preferences:* In addition to the prior point, information technology can go one step further. If accounting information systems become more customized, this could also apply to information retrieval per se. Practice and design-science based research might develop multidimensional database solutions for reporting purposes that can be aligned with individual preferences, e.g., with regard to measurement parameters, probability scenarios, and aggregation levels (Pellens et al. 1998, Gassen 2000; Pellens 2007). XBRL as well as the emerging European Single Electronic Format (ESEF) for financial data and the European Single Access Point (ESAP) for all other data are steps in this direction. Individualized information retrieval seems revolutionary, as it diminishes the need to standardize and to confine accounting and reporting that could, at best, be tailored to one stakeholder group. However, research has also to illuminate the other side of the coin: whether ordinary users or only well-trained specialist can finally understand and operate such a sophisticated tool, not to mention possible disadvantages for comparability as well as the costs of (big) data collection and verification, among other things (e.g., Fülbier et al. 2019).

Definition of the accounting entity for new organizational forms of economic activity: Digitalization gives rise to new, ‘agile’ organizational forms of economic activity between the market and the legal firm, which are emerging alongside traditional parent-subsidiary relationships. For example, special purpose entities, platform business models or open source communities are associated with high benefits, but ultimately also high costs and exploitation potential for some stakeholders. Possible manifestations include the invisible delegation of entrepreneurial responsibility to many small providers if companies see themselves only as contact platforms for supply and demand, the intransparent position of employees as self-liable entrepreneurs in ‘liquid organizations,’ and the question of liability if R&D activities are available to the public and other producers. Accounting research of all kinds might help to better understand and inform about these risks. A possible solution could be an intelligent organizational transparency which not only pictures the group, but also individual legal entities involved and their legal (intragroup) relationships (Fülbier and Gassen 2020). Another expansion of corporate reporting is already underway, with sustainability reporting requirements mandating firms to report information about economic risks and opportunities, as well as environmental and social impacts, emanating from their upstream and downstream value chains.

### Reorganizing and integrating corporate reporting

Financial reporting and management currently exist side by side with the emerging new data from sustainability reporting. Do we need hierarchies with one-dimensional summary measures such as variants of the traditional profit or loss, existing or new value-added figures, or completely new target figures from the financial or non-financial spheres?^28^ If so, what is the role of the shareholder and/or stakeholder approaches in this regard – do they compete here or merge into each other? At this point, there is already a veritable conceptual economic and legal discussion (e.g., Kaler 2003; Perrini and Tencati 2006; Wall and Greiling 2011; Bebchuk and Tallarita 2020; Mayer 2020) which by no means seems to be finished yet. Or is the future of corporate reporting and especially management accounting a multidimensional one with several reporting and management levels that cannot really be aggregated but, however, transferred into a few key figures, for example based on the balanced scorecard concept (e.g., Figge et al. 2002; Hansen and Schaltegger 2016; Kaplan and McMillan 2020)? A lot of more accounting and corporate reporting research of any kind seems necessary to clear the fog here.

## Where to start

In Sect. 2.2, we described what we perceive as researchers’ possible approach to selecting research projects by assessing private costs and benefits. Clearly, untenured junior scholars have little choice but to play by the ‘rules of the game’ as they seek to advance in their academic careers. As a result, some feel an immense pressure to publish—or perish. Working primarily for that extrinsic reward—the next publication—can feel shallow and stressful. It invites a focus on the type of research that ‘top journals’ and their gatekeepers seem to be looking for, which, as discussed above, may not always be the type that addresses important societal problems. In particular, since ‘wicked’ problems often require interdisciplinary efforts, young scholars without large networks to draw on, and who worry about the costs of interacting with researchers from other fields, face particular challenges.

The situation is slightly different for the tenured academics among us.^29^ Focusing primarily on trying to improve practice and society at large, regardless of publication output, has at least three advantages: First, addressing issues that we consider important for reasons other than getting published creates a sense of joy, flow, and purpose—which, in turn, makes us better and more productive. We could ask ourselves: ‘What am I doing for society?’, rather than, ‘Where is my work published and how many citations does it generate?’.

Second, there are economic reasons. During our careers, we have come across (increasingly, it seems) papers that, while technically superb, seem strangely sterile and disinterested in the accounting phenomenon under study. Some address questions that few practitioners would understand, much less care about (the research-practice gap, see above). Research without a ‘real impact’ on society that we can explain, at least in terms of long-term potential, may end up facing drying-up funds.^30^

And third, doing so may render academia more attractive again. The young people now embarking upon their careers, the corona, climate, and Ukrainian war crises firmly in mind, may be looking for purpose, meaning, and a chance to contribute to society, maybe more than any generation before them. Too many bright minds have been scared away from pursuing academic careers because of the prospect of the grueling tenure process—having to perform in a high-stakes, winner-takes-all publishing game with little regard for one’s true passions.

And the game may be rigged, too. Several recent scandals have uncovered manipulated research results in published papers in accounting and elsewhere. In a recent webinar, Jim Ohlson effectively argues that many accounting scholars do not care whether published results are correct. No wonder if (untenured) academics were to turn cynical about research. They may view pursuing research imbued with personal meaning and purpose as a distant dream—a utopia inhabited by the tenured. This is something that the ‘gatekeepers’ among us—the reviewers, editors, department chairs, recruiting and tenure committee members—need to change by re-defining the performance measures we use to assess young academics and the research they (and we) do. It seems that we need—to name just a few of the extremely complex challenges—a more tolerant methodological understanding (Feyerabend 1993; Fülbier and Weller 2008),^31^ more diverse and curiosity-oriented research (e.g., Hopwood 2007) with (sometimes, not always) less formalism and inclination towards the Cartesian ideal of quantitative, abstract research (e.g., Basu 2012; Dyckman and Zeff 2015), more interdisciplinary approaches, more education in the institutional environment including accounting and reporting skills and techniques, more focus on fundamental practical problems for business and society, more focus on teaching practitioners and policy makers with better communication and collaboration with them (e.g., Diamond 2005; Kaplan 2011; Basu 2012; Dyckman and Zeff 2015; Rajgopal 2020). We also need the courage to rearrange the current academic reward system to better motivate researchers to address problems of real importance (Fülbier et al. 2009), to rethink the process of academic publishing (e.g., Moizer 2009; Dyckman and Zeff 2015) and to reject simplistic, one-dimensional journal rankings in order to think again about proper instruments of research assessment (recently Fülbier and Ruhnke 2022). In consequence, we may be able to tap into new pools of brilliant, creative Ph.D. candidates and junior researchers, attract more non-academic funds, be prouder of our achievements as an academic community, and, last but not least, find advancing the language of business more enjoyable. Finally, however, attracting bright young minds will be greatly eased if there were more attractive positions available. Rather than forcing junior academics to compete for the few full professorships at German universities, which open up mostly when someone retires, we need positions that allow emerging scholars to conduct research and teaching under conditions of financial security and predictability. It is only under such conditions that passion and creativity can thrive.

## Data Availability

This paper is conceptual; hence, no data was used.

## Footnotes

1 For a critical discussion of its implications and limitations, see, for example, Bloomfield (2008).

2 According to Schnackenberg and Tomlinson (2016), disclosure of information describes the availability, accessibility, and visibility of information to receivers; accuracy of information describes the precision, reliability, and validity of the information disclosed by senders; and clarity of information describes the understandability and comprehensibility of the information to receivers.

3 Mattessich’s (1995a, 1995b) notion of “conditionally normative” accounting research summarizes this approach.

4 These include the German Stock Corporation Law (*Aktiengesetz*) of 1965, the German transposition of the EU Accounting Directive by the *Bilanzrichtliniengesetz* of 1985, as well as the legislation introducing internationally recognized accounting standards around the turn of the millennium.

5 For example, Leffson (1964), Baetge (1970), Küting (1974) or Moxter (1974); with a focus on more specific accounting rules, see for example von Keitz (1997) on intangibles or Leippe (2002) on leases.

6 By ‘top’ or ‘top-tier’ journals, we refer to the academic journals that are held in highest regard by the leading academic institutions in a field and geographic area, as identified by widely used (and heavily criticized) journal rankings. In German-speaking accounting academia, for example, the VHB-JOURQUAL 3 lists three journals in the top category (“A + ”): *The Accounting Review*, *Journal of Accounting and Economics*, and *Journal of Accounting Research*.

7 Kothari, Ramanna, and Skinner (2010) review the positive theory of GAAP and discuss the extent to which existing GAAP is consistent with the theory. Whereas earlier draft versions had the unapologetically normative title “What should GAAP look like?” (e.g., Kothari, Ramanna, and Skinner 2009), the published version contains the following disclaimer: “In summarizing the implications of extant research for GAAP, we run the risk of being interpreted as making normative prescriptions. That is not our intent. Our analysis is primarily positive in that it simply summarizes conclusions from the literature about those properties of GAAP that best facilitate the efficient allocation of capital” (p. 249). In what follows in that section of the article, it becomes clear that Kothari et al.’s (2010) approach is best summarized as Mattessich’s (1995a, 1995b) “conditional-normative accounting methodology.” Here, the researcher refers not to his/her own value system but to an externally given objective that is provided, for example, by superior law, conceptual frameworks, or empirical evidence. This research is prescriptive-normative as well, although the authors do not seem to recognize or admit it. Needless to say, we see no need for scholars to distance themselves from either approach.

8 Interestingly, the Journal of Accounting and *Economics* (emphasis added to make a point) is more highly ranked in the U.S. than the journal Accounting, Organizations and Society, which aims “to publish high quality work which draws upon diverse methodologies and theoretical developments from across the social sciences” (www.journals.elsevier.com/accounting-organizations-and-society).

9 An exception is perhaps, the event study method which arguably did originate in accounting research to some extent. “In the late 1960s seminal studies by Ray Ball and Philip Brown (1968) and Eugene Fama et al. (1969) introduced the methodology that is essentially the same as that which is in use today.” Yet: “Event studies have a long history. Perhaps the first published study is James Dolley (1933)” (MacKinlay 1997, pp. 13–14).

10 See the controversial debate about normative implications from positive findings surrounding value-relevance research in Barth et al. 2001, Holthausen and Watts 2001; see also Fülbier et al. (2009) about this.

11 Whereas the DFG-funded Collaborative Research Center (CRC; *Sonderforschungsbereich*) TRR 266 *Accounting for Transparency* (www.accounting-for-transparency.de) is a welcome success for the researchers involved, it represents a rare exception, being the first DFG CRC for business economics in Germany. However, the reasons underlying scarce competitive research funding for accounting and corporate reporting might be more complex, going beyond the chosen research questions and methods. The low amount of grants might also indicate lower funding needs in business and accounting, i.e., lower numbers of applications due to lower costs of conducting research compared to the natural sciences, medicine, and others. Additional factors could include the state funding system for institutional and also teaching-driven structures at the universities, as well as alternative funding from practice (probably with a greater focus on solving practical problems), which deserves much greater attention.

12 In order not to end this section on too somber a note, we again stress that our observations are necessarily subjective. It is far from certain that research and practice in accounting and corporate reporting would be in a “better” (however measured) state today had research taken a different route, and had therefore many thousand man-years of academics’ productive time been invested into a different mix of methods and theories.

13 These large, complex and pressing issues are called ‘wicked’ because they elude quick fixes and can only be addressed through concerted, interdisciplinary effort (Rittel and Weber 1973).

14 See, for example, Sellhorn and Wagner (2022a) and Großkopf, Sellhorn, Weiß, and Wagner (2021).

15 This is a view that is even shared by Earth system scientists, as evidenced by the keynote speech delivered by *Johan Rockström* at the European Accounting Association’s 2021 Annual Congress.

16 See, for example, the debate between Bebchuk and Tallarita (2020) and Mayer (2020), with a comment by Honold (2021).

17 This dominance, by the way, was never justified from a philosophy-of-science perspective (Fülbier and Weller 2008).

18 We are grateful to those who responded and provided us with thought-provoking insights and opinions.

19 “On the one hand, it takes the form of *compliance uncertainty* about which among several potential accounting treatments for the accounting issue in question meet the requirements. On the other hand, in selecting from the set of permitted accounting treatments, preparer managers consider the uncertain ‘payoffs’ associated with each accounting treatment – which reflect the information needs and preferences of various stakeholders, as well as managers’ economic incentives, legitimacy and reputation concerns, compliance costs, and other objectives (*outcome uncertainty*)” Hombach and Sellhorn (2022, p. 3).

20 For more examples and an in-depth discussion of what constitutes an unresolved accounting issue, we refer the reader to Hombach and Sellhorn (2022) and Schipper (2022).

21 The difference between ‘rolling’ and ‘frozen GAAP’ is explained in Leftwich (1983).

22 For example, Krause, Ahmed, and Sellhorn (2017).

23 See, for example, Johnson and Petrone (1998) and Sellhorn (2004).

24 The IASB has noted the former paper during its PIR, as evidenced by the respective literature review staff paper dated July 2021 (Agenda ref 7D), although it is not clear from public IASB documents whether and how its findings have shaped the IASB’s conclusions.

25 For example, when it comes to combatting the carbon emissions that underlie the climate crisis, three regulatory instruments are available: (1) command-and-control regulation, i.e., requirements (e.g., for home owners to install solar panels) and bans (e.g., of coal-fired power plants, or combustion engines in cars) supplemented with fines or other penalties for violation; (2) financial incentives, such as a carbon tax or a carbon-emissions trading scheme, which harness the market mechanism to achieve emissions reductions more efficiently, i.e., where they are least costly to achieve; and (3) targeted transparency regulation, such as mandatory, audited and enforced public disclosure of carbon emissions and other ESG information – the idea being that forcing firms to disclose their climate-related impact and exposure will unleash responses from shareholders and other stakeholders that will discipline firms to cut emissions.

26 Joachim Gassen, for example, talked recently at the Ruhr-University Bochum about this issue; he used the pointed title: “Should accountants change the world? On the role of accounting research and professional practice” (FAACT Seminar, July 2022).

27 See www.accounting-for-transparency.de.

28 A series of articles in the *Frankfurter Allgemeine Zeitung* controversially discussed the challenge to identify key performance indicators for accounting, reporting and especially management purposes. For example, see Honold (2020), Hutzschenreuter (2021), Simon (2020), Wagner (2020), Weißenberger (2020).

29 For much of the following, see Sellhorn (2020).

30 To some, this argument may appear like wishful thinking. The alternative story, where societal demand for more diverse research may *not* translate into changing university preferences and incentive systems, is about as follows. University leaders do not usually perceive accounting academics as research active, and hence they have low expectations (“demand”) in this regard. Accounting academics are not usually expected to acquire third-party research funding, and hence often do not, and whatever we do acquire is little compared to what other fields do. Rather, university leaders need accounting academics to teach the large numbers of business and economics students. It is our view that, to remain attractive to these students, our teaching needs to be relevant to today’s societal problems, and since our research feeds our teaching, so does our research.

31 This is especially true of the gatekeepers among us. TAR is a good example in every respect. While Ball and Brown (1968) was rejected there as an “out-of-the-box paper”, a decade later the editor Stephen Zeff, according to his own statement, managed to find tolerant and open-minded reviewers for the Watts and Zimmerman (1979) paper about the market for excuses, which was ultimately an attack against the still-important normative academia in the time. It remains an open question whether this would be the case today with “out-of-the-box papers”.
